# Eighteen-year trajectories of depressive symptoms in mothers with a lifetime eating disorder: findings from the ALSPAC cohort

**DOI:** 10.1192/bjp.2019.89

**Published:** 2020-02

**Authors:** Yu Wei Chua, Gemma Lewis, Abigail Easter, Glyn Lewis, Francesca Solmi

**Affiliations:** 1 PhD candidate in Education, Laboratory for Innovation in Autism, University of Strathclyde, UK; 2 Research Associate in Psychiatric Epidemiology, Division of Psychiatry, University College London, UK; 3 Senior Postdoctoral Research Fellow, Centre for Implementation Science, Institute of Psychiatry, Psychology and Neuroscience, King's College London, UK; 4 Professor of Epidemiological Psychiatry, Division of Psychiatry, University College London, UK; 5 Sir Henry Wellcome Post-doctoral Fellow, Division of Psychiatry, University College London UK

**Keywords:** ALSPAC, eating disorders, depression, parental mental health, perinatal mental health

## Abstract

**Background:**

Two longitudinal studies have shown that depressive symptoms in women with eating disorders might improve in the antenatal and early postnatal periods. No study has followed up women beyond 8 months postnatal.

**Aims:**

To investigate long-term trajectories of depressive symptoms in mothers with lifetime self-reported eating disorders.

**Method:**

Using data from the Avon Longitudinal Study of Parents and Children and multilevel growth curves we modelled trajectories of depressive symptoms from the 18th week of pregnancy to 18 years postnatal in women with lifetime self-reported anorexia nervosa, bulimia nervosa or both anorexia and bulimia nervosa. As sensitivity analyses we also investigated these trajectories using quintiles of a continuous measure of body image in pregnancy.

**Results:**

Of the 9276 women in our main sample, 126 (1.4%) reported a lifetime diagnosis of anorexia nervosa, 153 (1.6%) of bulimia nervosa and 60 (0.6%) of both anorexia and bulimia nervosa. Women with lifetime eating disorders had greater depressive symptoms scores than women with no eating disorders, before and after adjustment for confounders (anorexia nervosa: 2.10, 95% CI 1.36–2.83; bulimia nervosa: 2.28, 95% CI: 1.61–2.94, both anorexia and bulimia nervosa: 2.86, 95% CI 1.81–3.90). We also observed a dose–response association between greater body image and eating concerns in pregnancy and more severe trajectories of depressive symptoms, even after adjusting for lifetime eating disorders which also remained independently associated with greater depressive symptoms.

**Conclusions:**

Women with eating disorders experience persistently greater depressive symptoms across the life-course. More training for practitioners and midwives on how to recognise eating disorders in pregnancy could help to identify depressive symptoms and reduce the long-term burden of disease resulting from this comorbidity.

There is evidence that women with eating disorders, such as anorexia nervosa and bulimia nervosa, experience more postnatal (i.e. up to 12 months after pregnancy) depressive symptoms than the general population. Clinical studies have found that up to a third of women with eating disorders report postnatal depression^1–3^ and that, among women who experienced both anorexia nervosa and bulimia nervosa over the course of their lifetime, this proportion increased to two-thirds.^2^ Investigations of general population samples have shown that between 40% and 66% of women with a history of eating disorders had postnatal depression,^4–6^ compared with 5–13% (depending on severity of definition) in the general population.^7^ Existing evidence on postnatal depression in women with eating disorders has several limitations. First, many studies use clinical samples – some without a healthy comparison group^1–3^ – which can lead to selection bias and, potentially to an overestimation of this comorbidity. Second, most clinical and population-based studies use small samples, possibly with low statistical power.^1–3,5,6^ Third, studies have rarely investigated whether postnatal depressive symptoms vary by eating disorder diagnoses^6^ opting for broader definitions of current or past eating disorders^5,8^ or focusing on bulimia nervosa.^2,3^ Understanding whether these associations vary by eating disorder diagnoses can help to inform treatment approaches and shed light on shared or specific aetiological factors. Finally, the majority of studies used cross-sectional designs that do not allow study of how depressive symptoms develop over time.^2,3,6^

Two longitudinal studies have explored the trajectory of postnatal depressive symptoms in mothers with eating disorders but only followed participants to 8 months postnatal.^5,8^ These studies show that, overall, women with current or past eating disorders experience a reduction in symptoms during pregnancy,^5,8^ but that for those with current eating disorders their symptoms might increase in the postnatal period.^8^ As far as we are aware, no study has investigated longer-term trajectories of depressive symptoms in mothers with lifetime eating disorders. This would provide important information on the longer-term mental healthcare needs of this population. We investigated trajectories of depressive symptoms in mothers who reported a history of anorexia nervosa, bulimia nervosa or both, from the 18th week of pregnancy up to when their child was 18 years of age. As sensitivity analyses, we also explored these trajectories in women with greater body image and eating concerns in pregnancy, adjusting for lifetime eating disorder diagnosis. This allowed us to test whether current body image and eating concerns and past self-reported diagnoses were independently associated with depression trajectories.

## Method

### Participants

We used data from the Avon Longitudinal Study of Parents and Children (ALSPAC), a population sample of pregnant women. A total of 14 541 women resident in the former country of Avon (UK) with an expected delivery date between 1 April 1991 and 31 December 1992 were invited to take part in the study.^9^ Of the 14 062 children (96.7% of all births) included in the study, 13 988 (99.5% of all live births) were alive at 1 year of age. The study website (www.bristol.ac.uk/alspac) provides more details on the sample.^9,10^ The study website also contains details of all the data that is available through a fully searchable data dictionary available at: http://www.bris.ac.uk/alspac/researchers/data-access/data-dictionary/. ALSPAC mothers, their children and partners have been followed up since recruitment with postal questionnaires and clinical assessments, including biological samples.

In this study, we included women with complete data on our exposure and confounders, who also had depressive symptoms measured on at least three follow-up assessments. The cut-off of three assessments was chosen to ensure reliability of trajectory estimation. Ethical approval for the study was obtained from the ALSPAC Law and Ethics committee and the local research ethics committees.

### Exposure

#### Main analyses

History of lifetime eating disorder was assessed via self-report at 12 weeks’ gestation. From individual questionnaire items (see supplementary Appendix 1 available at https://doi.org/10.1192/bjp.2019.89) we derived a categorical variable indicating: no eating disorder, anorexia nervosa, bulimia nervosa or both anorexia and bulimia nervosa. This variable has been previously used in this sample^11^ as there is evidence that self-reported eating disorders can be a valid measure of lifetime diagnoses.^12^

#### Sensitivity analyses

At 18 weeks’ gestation women were asked ten questions (scored on a Likert scale 0, not at all; 1 yes, sometimes; 2, yes, mostly) developed by Fairburn & Stein to measure body image dissatisfaction and attitudes to eating in pregnancy (Supplementary Appendix 1; C Fairburn & A Stein, personal communication, 1990). Total scores were divided in quintiles (first quintile: lower cognitions; fifth quintile: higher cognitions).

### Outcome

We assessed depressive symptoms using the Edinburgh Postnatal Depression Scale (EPDS), a 10-item self-report measure of depressive symptoms.^13^ The EPDS has been extensively used to measure parental depression in general population studies and has been validated beyond the postnatal period.^13^ Possible scores range from 0 to 30 with higher scores indicating greater depressive symptoms.^14^ Depressive symptoms were measured at nine time points: at 18 and 32 weeks’ gestation; at 8 weeks and 8 months postnatally; and when the children were approximately 2, 3, 5, 6, 8, 11 and 18 years of age.

### Confounders

Potential confounders of the association between eating disorders and postnatal depressive symptoms were identified as: age at delivery, maternal social class (non-manual/manual), highest educational qualification achieved (compulsory/non-compulsory), and whether at least one parent (of the mother) had a diagnosis of any mental disorder (yes/no). We further included history of sexual abuse as a potential confounder given its known associations with both eating disorders and depression. Other than age at delivery, these variables were assessed using self-report questionnaires at 32 weeks’ gestation.

### Data analysis

We described our sample in relation to exposure, confounders and outcome distributions using cross-tabulations with proportions and means with standard deviations, testing for overall group differences with one-way χ^2^ tests and ANOVAs, respectively. We investigated the correlation between depressive symptoms across follow-up times.

We used multilevel modelling with growth curves (repeated depression measurements nested within individuals) to investigate the association between lifetime eating disorder diagnoses and depressive symptom trajectories, using full information maximum likelihood estimation to include those with at least three outcome measurements. First, we modelled trajectories of depressive symptoms across time with a linear slope term (time) (model A). In model B, we further included a quadratic slope term (time^2^) and random effects for both time and time^2^ as this provided a better fit for the data (see supplementary Table 1). Next, we fitted multivariable conditional models. In model C, all confounders were included as fixed effects. Lifetime eating disorder diagnoses were added as a fixed effect in model D. Finally, in model E, we included interaction terms between lifetime eating disorder diagnoses and linear time, and lifetime eating disorder diagnoses and quadratic time. This tested differences in the slope of depressive symptom trajectories over time. In all models child age was mean centred at 4.8 years. We chose 4.8 years as it represented the mean follow-up time point and hence we hypothesised it would provide an average estimate of group differences (i.e. model intercept) over time.

To explore the potential for missing data to bias our results, we ran our models on women with complete exposure data and at least three outcome measurements, and imputed missing confounder and outcome data. We imputed 50 data-sets using multiple imputation by chained equations with linear, logistic and multinomial logistic regression models.^15^ As recommended in the literature,^16^ our imputation models included all variables used in our main analyses, plus a number of auxiliary variables: parity, marital status and smoking during pregnancy. As a sensitivity check, we further ran our complete cases models (based on those women with complete exposure and confounder data) and at least one outcome measurement available in order to further explore whether any bias was introduced by restricting our analyses to women with more follow-up measurements.

In sensitivity analyses, we tested whether body image and eating concerns in pregnancy were higher in women with lifetime eating disorder, using univariable and multivariable (adjusting for maternal age, social class and education) linear regression models with total eating disorder cognition score as our outcome. Further, we investigated trajectories of depressive symptoms across quintiles of body image and eating concerns in pregnancy using multilevel growth curves and the same approach to sample definition and model building as described above, further adjusting for lifetime eating disorders.

## Results

### Participants

A total of 12 411 women had complete exposure data and, of these, 11 590 (93.4%) had at least three EPDS measurements. Of the latter, 2314 (20.0%) had missing confounder information, leaving 9276 women in our complete case sample (74.7% of those with complete exposure, 80.0% of those with complete exposure and outcome).

As shown in Table 1, in our complete case participants 126 (1.4%) women had a history of anorexia nervosa, 153 (1.6%) of bulimia nervosa, and 60 (0.6%) of both anorexia and bulimia nervosa. Most women had non-manual occupations (81.0%) and had completed compulsory education (59.4%). The average age at delivery was 28.6 years (s.d. = 4.7 years). Less than 5% of women reported a history of parental mental disorder (4.4%) or sexual abuse (4.7%). Compared with women without a history of eating disorders, those with a lifetime diagnosis of anorexia nervosa, bulimia nervosa, or both had greater educational attainment, and were more likely to not be married and to have a history of parental mental health problems or sexual abuse (Table 1).

**Table 1:** Participants characteristics

	Participants, total	No eating disorder	Anorexia nervosa	Bulimia nervosa	Anorexia nervosa + bulimia nervosa	*P*
Lifetime eating disorder, *n* (%)	9276 (100)	8937 (96.3)	126 (1.4)	153 (1.6)	60 (0.6)	
Social class, *n* (%)						
Manual	1763 (19.0)	1692 (18.9)	25 (19.8)	33 (21.6)	13 (21.7)	0.80
Non-manual	7513 (81.0)	7245 (81.1)	101 (80.2)	120 (78.4)	47 (78.3)	
Education, *n* (%)						
Compulsory	5513 (59.4)	5348 (59.8)	59 (46.8)	82 (53.6)	24 (40.0)	<0.001
A level or degree	3763 (40.6)	3589 (40.2)	67 (53.2)	71 (46.4)	36 (60.0)	
Parental mental disorder, *n* (%)						
No	8866 (95.6)	8562 (95.8)	114 (90.5)	138 (90.2)	52 (86.7)	<0.001
Yes	410 (4.4)	375 (4.2)	12 (9.5)	15 (9.8)	8 (13.3)	
History of sexual abuse, *n* (%)						
No	8844 (95.3)	8547 (95.6)	113 (89.7)	137 (89.5)	47 (78.3)	<0.001
Yes	432 (4.7)	390 (4.4)	13 (10.3)	16 (10.5)	13 (21.7)	
Age at delivery, years: mean (s.d.)	28.6 (4.7)	28.6 (4.7)	29.4 (5.1)	28.2 (4.5)	29.3 (3.9)	0.11

### Missing data

Among women with complete exposure data, 812 (6.5%) had fewer than three follow-up EPDS measurements. As shown in supplementary Table 1, women with a history of anorexia nervosa, who were younger, unmarried, with a manual occupation, and who had only completed compulsory education and smoked in pregnancy were more likely to have missing outcome data.

### Lifetime eating disorders and depressive symptoms

As shown in supplementary Table 2, in the complete case participants, mean depressive symptoms scores ranged, across time points, between 5.2 (s.d. = 4.5) at 8 months' postnatal to 7.4 (s.d. = 5.4) at 18 years of age of the offspring. On average women had greater depressive symptoms during pregnancy (18 weeks' gestation mean 6.7 (s.d. = 4.7), 32 weeks' gestation mean: 6.8 (s.d. = 4.9)) and at the offspring's 18th year of age (mean 7.4, s.d. = 5.4). Depressive symptoms were moderately to highly correlated between each time point, and more strongly correlated for measurements made closer in time, as we would expect in repeated measures designs (supplementary Table 3). At all time points, women with lifetime eating disorders had greater depressive symptoms supplementary Table 2).

### Growth curve models

**Fig. 1:**
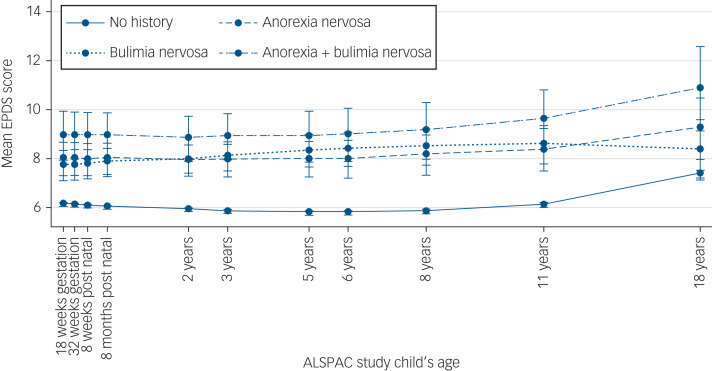
Predicted trajectories of depressive symptoms derived from model E by self-reported lifetime eating disorder.

EPDS, Edinburgh Postnatal Depression Scale; ALSPAC, Avon Longitudinal Study of Parents and Children.

In supplementary Table 4 we report our model-building approach for unconditional models A and B and conditional model C, and in Table 2, results of models D and E. In model D, we found evidence that women with lifetime anorexia nervosa, bulimia nervosa, and both anorexia and bulimia nervosa all had greater depressive symptoms (anorexia nervosa coefficient: 1.88, 95% CI 1.24–2.51; bulimia nervosa coefficient: 1.72, 95% CI 1.15–2.30; both anorexia and bulimia nervosa coefficient: 2.68, 95% CI 1.77–3.60). The overlapping 95% CIs suggest that these differences did not vary by group. In model E, where we included two interaction terms between eating disorders and time and eating disorders and time^2^, results were largely unchanged with self-reported eating disorder still reporting greater depressive symptoms, regardless of diagnosis, as can be seen in Fig. 1 plotting the model estimates. When we relaxed our assumptions on outcome missingness and extended the sample to women with at least one outcome measurement available results were comparable (see supplementary Table 5).

**Table 2:** Results of multilevel models D and E showing the association between maternal lifetime eating disorder and trajectories of depressive symptoms in the complete case participants (*n* = 9276)^a^

	Depressive symptoms	
	Model D, coefficient (95% CI)	Model E, coefficient (95% CI)
Lifetime eating disorder		
No eating disorder	Reference	Reference
Anorexia nervosa	1.88** (1.24 to 2.51)	2.10** (1.36 to 2.83)
Bulimia nervosa	1.72** (1.15 to 2.30)	2.28** (1.61 to 2.94)
Anorexia nervosa + bulimia nervosa	2.68** (1.77 to 3.60)	2.86** (1.81 to 3.90)
Lifetime eating disorder × time		
No eating disorder	–	Reference
Anorexia nervosa	–	0.0034 (−0.0034 to 0.010)
Bulimia nervosa	–	0.0073* (0.0012 to 0.013)
Anorexia nervosa + bulimia nervosa	–	0.0054 (−0.0040 to 0.015)
Lifetime eating disorder × time^2^		
No eating disorder	–	Reference
Anorexia nervosa	–	−0.00004 (−0.00010 to 0.00003)
Bulimia nervosa	–	−0.00011** (−0.00017 to −0.00005)
Anorexia nervosa + bulimia nervosa	–	−0.00002 (−0.00011 to 0.00007)

a. Model D: adjusting for maternal age, education, social class, history of sexual abuse and parental mental health. Model E: adjusting for model D + interactions between maternal eating disorder and both time and time square.

* 0.01 > *P* ≥ 0.0001; **P < 0.0001.

### Multiple imputation

We also re-ran models A–E in the those women (*n* = 11 590) with complete exposure data and three or more depression measurements, and imputed missing confounders and outcome. As can be seen in supplementary Table 6, results did not change substantially in these models. Women with a lifetime eating disorder still had greater postnatal depressive symptoms in models D and E and, although coefficients were larger in the imputed sample, 95% CIs largely overlapped with those of our main analytical samples.

### Sensitivity analyses

A total of 8722 women had complete data on body image and eating concerns in pregnancy, at least three outcome measurements, and all confounders including maternal self-reported eating disorder diagnosis. Of these, 2316 (26.6%), 1823 (20.9%), 1524 (17.5%), 1569 (18.0%), and 1490 (17.0%) were in the first, second, third, fourth and fifth quintile of body image and eating concerns, respectively. Compared with those in the bottom quintile, more women in the top quintile reported a history of sexual abuse (3.6% *v.* 7.7%) and parental mental health problems (3.7% *v.* 5.6%). Women in the top quintile were also younger (28.4 *v.* 28.7 years) (data available from the authors on request). Women with lifetime self-reported eating disorder reported greater body image dissatisfaction and eating concerns in pregnancy (adjusted coefficients for anorexia nervosa: 1.07, 95% CI 0.30–1.94; bulimia nervosa: 2.83, 95% CI 2.14–3.51, both anorexia and bulimia nervosa: 2.89, 95% CI 1.80–2.98, see supplementary Table 7).

Results of model A–C are reported in supplementary Table 8. Table 3, shows the results of models D and E. In both models, we observed a dose–response association between increasing levels of body image dissatisfaction and eating concerns and depressive symptoms (see also supplementary Fig. 1). For women in the top quintile of body image and eating concerns, depressive symptoms appeared to decrease at a higher pace in the antenatal and postnatal periods, although remaining the most symptomatic group over the entire follow-up period. Lifetime eating disorder remained very strongly associated with greater depressive symptoms independently of current symptoms with coefficients comparable with those observed in the main analyses.

**Table 3:** Results of multilevel models D and E showing the association between quintiles of maternal body image and eating concerns in pregnancy and trajectories of depressive symptoms in complete case participants (*n* = 8722)^a^

	Depressive symptoms	
	Model D, coefficient (95%CI)	Model E, coefficient (95%CI)
Quintiles of eating disorder cognitions		
1st (lowest)	Reference	Reference
2nd	0.75 (0.55 to 0.97)**	0.68 (0.43 to 0.92)**
3rd	1.20 (0.98 to 1.42)**	1.15 (0.89 to 1.41)**
4th	1.94 (1.72 to 2.15)**	1.78 (1.52 to 2.04)**
5th (highest)	3.23 (3.01 to 3.46)**	2.88 (2.62 to 3.14)**
Quintiles of eating disorder cognitions × time		
1st (lowest)	–	Reference
2nd	–	−0.001 (−0.003 to 0.002)
3rd	–	−0.001 (−0.004 to 0.001)
4th	–	−0.003 (−0.005 to −0.001)*
5th (highest)	–	−0.006 (−0.009 to −0.004)**
Quintiles of eating disorder cognitions × time^2^		
1st (lowest)	–	Reference
2nd	–	0.00002 (−0.00001 to 0.0003)
3rd	–	0.00001 (−0.00002 to 0.00003)
4th	–	0.00002 (−0.00001 to 0.00005)
5th (highest)	–	0.00005 (0.00003 to 0.00008)**
Lifetime eating disorder		
No eating disorder	Reference	Reference
Anorexia nervosa	1.50 (0.86 to 2.13)**	1.49 (0.86 to 2.13)**
Bulimia nervosa	1.16 (0.59 to 1.73)**	1.16 (0.59 to 1.73)**
Anorexia nervosa + bulimia nervosa	1.84 (0.94 to 2.74)**	1.84 (0.94 to 2.74)**

a. Model D: adjusting for maternal age, education, social class, history of sexual abuse, parental mental health and maternal self-reported lifetime eating disorder. Model E: adjusting for model D + interactions between maternal body image and eating concerns in pregnancy and both time and time square.

*0.05 ≥ *P* ≥ 0.01; **P < 0.0001.

## Discussion

In this large longitudinal general population cohort, we found that women with an eating disorder at some point in their lives had greater depressive symptoms in the perinatal period and that these symptoms persisted over an 18-year follow-up. We also found that women with greater body image and eating concerns in pregnancy had higher depressive symptoms across the life-course, after accounting for self-reported lifetime eating disorder, which was still independently associated with the outcome.

### Strengths and limitations

To our knowledge, this is the first study to explore long-term (i.e. from pregnancy until age 18 of their study child) trajectories of depressive symptoms among mothers with lifetime eating disorders and current eating disorder symptoms. This study had a number of strengths. We used data from a large general population cohort of mothers, which allowed us to avoid biases associated with clinical samples and investigate symptom presentations that might be common in the general population. Owing to the large size of our sample we could also investigate differences in patterns of depressive symptoms across eating disorder diagnoses, which have not been explored in previous literature. Finally, our multilevel analytical approach allowed us to make efficient use of the longitudinal nature of our sample and minimise the impact of losses to follow-up.

Our study also had a number of limitations. First, our main exposure variable was based on a self-reported measure of lifetime eating disorder, which could have resulted in some degree of misclassification or recall bias. However, a number of studies have used this definition^11,17^ as there is evidence that this approach yields acceptable levels of sensitivity and specificity in community surveys.^12^ Our prevalence estimates are also in line with those previously observed in the literature.^18^ the use of lifetime data allows the investigation of relatively uncommon disorders such as eating disorders, which are understudied in population-based cohorts.

Women were asked whether they had ever had anorexia or bulimia nervosa. For this reason, we might have missed women with atypical presentations, binge eating disorder – which was not considered as a full eating disorder in DSM-IV – or individuals with cases of undiagnosed anorexia nervosa and bulimia nervosa (i.e. that women might have been unable to recognise as such). To tackle this limitation, we also used a measure of current symptoms, as opposed to diagnoses and not only did we find comparable results, but also observed that the two exposures were strongly and independently associated with our outcome.

Because eating disorders are relatively uncommon, despite our large sample size and the use of lifetime diagnoses, our main exposure categories were not very large, particularly for those with both anorexia and bulimia nervosa resulting in broad 95% CIs often overlapping between groups. A previous study^2^ found that women with a history of both anorexia and bulimia nervosa reported twice as many cases of postnatal depression compared with women with bulimia nervosa only, lending support to our finding of greater symptom severity in this group.

The associations we observed in our sensitivity analyses could be the result of reverse causation, so that women with greater depressive symptoms expressed more concerns about their image and eating. Similarly, given the self-reported and retrospective nature of the exposure, it was not possible to disentangle the relative contribution of eating disorders and lifetime depression to the outcome, nor the direction of this association. Regardless, we believe that this association offers important insight on the coexistence of these symptoms in pregnancy and might help to identify women at greater risk for both conditions in the short and long term.

### Comparison with previous literature

Our findings are largely consistent with those of previous investigations using a trajectory-based modelling approach, finding that women with current or lifetime history of eating disorders have greater depressive symptoms compared with the general population.^5,8^ These studies, however, also showed that depressive symptoms in women with eating disorders decrease over the antenatal and perinatal period.^5,8^

In line with these findings, we observed that women in the top quintile of current body image and eating concerns in pregnancy experienced some degree of symptom improvement over time (see Supplementary Figure 1), although these were not sustained over time. Our findings suggest that these previously observed improvements in depressive symptoms over the antenatal and perinatal periods could be temporary. Alternatively, we suggest that these discrepancies between our findings and those of previous investigations could have emerged because of differences in exposure definition.^5,8^

First, we did not define eating disorders as current or past because we would not have had enough statistical power to investigate patterns of depression in women who reported anorexia nervosa or bulimia nervosa to have occurred ‘recently’ (see Supplementary Appendix 1). Nevertheless, to better understand the role of current eating disorder symptoms, we also explored trajectories of depressive symptoms according to current body image and eating concerns while adjusting for lifetime eating disorder. Here, we found that both were independently associated with greater depressive symptoms. This suggests that both lifetime diagnoses and current body image concerns could help to identify women at increased risk of depression not just in pregnancy, but also over the life-course.

Second, we did not group eating disorders according to presence or absence of self-reported lifetime diagnosis of ‘severe depression’. Given the retrospective nature of the questions included in ALSPAC, we would not have been able to exclude that depression was a consequence of eating disorders. Hence, stratifying for depression could have masked the effect of the exposure on the outcome. Nevertheless, future studies should further explore mechanisms that explain these patterns of comorbidity, as they could provide important insights on the aetiology of these conditions across the life-course.

### Interpretation of findings

The finding that mothers with a lifetime history of eating disorders – regardless of their diagnosis – continue to experience greater depressive symptoms up to 18 years after pregnancy could indicate that a considerable number of women might not fully recover from eating disorders, since these are highly comorbid with depression. It is known that, among women treated for eating disorders, around a third do not fully recover.^19^ A study by Bardone-Cone and colleagues found that women who achieve only partial recovery from eating disorders scored similarly to women with active eating disorders on a number of eating disorder, body image and psychopathology scales (including depression and anxiety) with greater scores compared with healthy controls and women who had achieved full recovery.^20^

However, we also found that lifetime eating disorders were still associated with greater depressive symptoms, regardless of body image and eating concerns in pregnancy. This association is consistent with the hypothesis that depressive symptoms might be a risk factors for onset of eating disorders and persist after recovery. Supporting this hypothesis, a study of adolescents who had achieved a 3-year recovery from anorexia nervosa (defined as weight restoration, stable periods, and absence of eating disorder symptoms) found that the latter, compared with healthy controls, still reported greater obsessive–compulsive, anxious and depressive traits.^21^

The potential persistence of depressive symptoms in mothers after eating disorder recovery is of note, as the former have been associated with negative outcomes in the offspring. A previous study has shown that children of women with eating disorders had greater odds of mental health difficulties in early childhood and that these associations were largely mediated by the presence of depression and anxiety in pregnancy.^22^ Although little is known about the long-term psychopathological outcomes of children of mothers with eating disorders, postnatal depression has consistently been associated with offspring developmental delays,^23^ higher rates of psychopathology^24^ and obesity.^25^ A recent study has also shown that children of mothers with more severe and persistent maternal depression,^26^ have adverse long-term outcomes, including greater depression and poorer academic achievement. This evidence suggests that children of women with eating disorders could be at greater risk of a number of adverse outcomes by way of the latter experiencing greater and more persistent depressive symptoms. Future studies should explore these associations.

Finally, it is worth noting that for all women, regardless of eating disorder diagnosis, depressive symptoms increased when their offspring was 18 years of age. Recent nationwide UK data from the 2014 Adult Psychiatry Morbidity Survey found that the prevalence of depression was highest in women aged 16–24 years, decreased between 25 and 44 and then increased again between the ages of 45 and 54.^27^ The reasons for the increase in depression between the ages of 45 and 54 years are poorly understood, but they likely involve a complex interplay of social, psychological and biological factors. The menopause, for example, occurs between the ages of 44 and 55 years, with an average age in the UK of 51 years. Depressive symptoms have been found to increase during the menopausal transition (around 3 years prior to the menopause).^28^ More research on risk factors for midlife depression in women are thus warranted.

To conclude, from a public health point of view, identifying pregnant women who have had an eating disorder in the past or might be experiencing concerns with their body image in pregnancy could help to also detect women at risk for depression. This might help to reduce the burden of postnatal depression, with benefits for both women and, potentially, their offspring. High levels of stigma perceived by people with eating disorders and difficulties in identifying eating disorders in general practice represent known barriers to treatment access, particularly in pregnancy.^29^ A recent systematic review suggests that presence of mental health comorbidity is perceived by people with eating disorders as facilitating help-seeking.^30^ In the UK, current guidelines from the National Institute for Health and Care Excellence (NICE) recommend using the EPDS or the Whooley questionnaire as part of a general discussion about woman's mental health and well-being during pregnancy and in the early postnatal period.^31^ As recent evidence confirms, women who disclosed depressive symptoms using these measures were also more likely to report other comorbid mental health disorders, including eating disorders.^32^ Hence, identifying depression in women with current or past eating disorders could provide them with a gateway to accessing more intensive perinatal care, as recommended by NICE.^33^ This could also help to prevent postnatal relapse of the eating disorder and long-term depressive symptoms identified in this study.

It is of critical importance to increase awareness of eating disorders in pregnancy and to train clinicians and midwives to recognise and support women with eating disorders in this crucial phase of their lives. The antenatal and early postnatal periods can be a time when eating disorder symptoms temporarily improve^34^ and women might be more open to change, hence they could be a key moment for the identification of these conditions with long-term benefits for these women.

## Supplementary material

For supplementary material accompanying this paper visit https://doi.org/10.1192/bjp.2019.89. click here to view supplementary material
