# Supplementary material for: Eighteen-year trajectories of depressive symptoms in mothers with a lifetime eating disorder: findings from the ALSPAC cohort
Source: Br J Psychiatry. 2019 May 14;216(2):90–6. doi: 10.1192/bjp.2019.89 (PMC7557597; doi:10.1192/bjp.2019.89)

**SUPPLEMENTAL MATERIAL**

**Exposure measures**

Lifetime eating disorders: Women were asked whether they had ever experienced AN (question: “Have you ever had any of the following problems: anorexia nervosa”) and BN (question: “Have you ever had any of the following problems: bulimia nervosa”). Possible answers were: ‘never’, ‘yes, in the past’, ‘yes, recently’, or ‘don’t know’. Because only a minority of women reported recent EDs (AN n=7, 0.06%, BN n=55, 0.46%) we grouped them with those who said ‘yes in the past’ to create a binary ‘yes/no’ lifetime category. From these two binary questions, we then derived a four-level categorical variable indicating: no ED history, lifetime AN; lifetime BN, or lifetime reports of both AN and BN. Women who said they did not know, were coded as missing data.

Body image and eating concerns in pregnancy were measured using 10 questions measured on a Likert scale (0 = not at all, 1= yes, sometimes, 2= yes, mostly) designed by Fairburn and Stein. These questionnaire were:

1. Has thinking about your shape or weight interfered with your ability to concentrate on things?
2. Have you been afraid that you may become fat?
3. Have you felt fat?
4. Have you had a strong desire to lose weight?
5. Has your weight influenced the way you think about yourself as a person?
6. Have you felt dissatisfied with your weight?
7. Have you felt dissatisfied about your shape?
8. Have you felt concerned about other people seeing you eat?
9. Have you felt uncomfortable seeing your body in the mirror?
10. Have you experienced a loss of control over eating?

**Supplemental Table 1: Univariable logistic regression models testing the association between model and MI auxiliary variable and outcome missingness**

| **Variables** | **<3 EPDS measurements,**  **N=812**  **OR (95%CI)** |
| --- | --- |
| **ED history** |  |
| No ED | Ref |
| AN | 1.63 (0.99 – 2.66), p=0.05 |
| BN | 0.89 (0.50 – 1.61), p= 0.71 |
| AN-BN | 0.92 (0.37 – 2.29), p=0.86 |
| **Social Class** |  |
| Manual | Ref |
| Non-manual | 0.56 (0.42 – 0.73), p<0.0001 |
| **Education** |  |
| Compulsory | Ref |
| A Level or degree | 0.37 (0.28 – 0.47), p<0.0001 |
| **Parental mental disorder** |  |
| No | Ref |
| Yes | 0.77 (0.45 – 1.33), p=0.35 |
| **History of sexual abuse** |  |
| No | Ref |
| Yes | 1.56 (1.07 – 2.27), p= 0.02 |
| **Marital status** |  |
| Married | Ref |
| Single (never married) | 2.34 (1.97 – 2.77). p<0.0001 |
| Widowed, divorced, separated | 2.06 (1.56 – 2.72), p<0.0001 |
| **Parity** |  |
| First child | Ref |
| Second or later | 1.08 (0.92 – 1.26), p=0.38 |
| **Smoking during pregnancy** |  |
| No | Ref |
| Yes | 2.23 (1.89 – 2.62), p<0.0001 |
| **Age, years** |  |
|  | 0.90 (0.89 – 0.91), p<0.0001 |

***** The analytical sample is further reduced by missingness in confounding variables.

**Supplemental table 2: Mean depression scores (Edinburgh Post Natal Depression) at each follow up time point, by eating disorder group**

|  | **Depressive symptoms** | | | | | | | | | |
| --- | --- | --- | --- | --- | --- | --- | --- | --- | --- | --- |
| **Time point** | **Overall** | | **No ED** | | **AN** | | **BN** | | **AN/BN** | |
|  | **N** | **Mean**  **(SD)** | **N** | **Mean**  **(SD)** | **N** | **Mean**  **(SD)** | **N** | **Mean**  **(SD)** | **N** | **Mean**  **(SD)** |
| **18w gest** | 8746 | 6.6(4.7) | 8430 | 6.5(4.6) | 116 | 8.0(5.0) | 143 | 8.0(4.7) | 57 | 8.9(5.2) |
| **32w gest** | 9216 | 6.7(4.9) | 8878 | 6.6(4.9) | 126 | 8.1(5.8) | 152 | 8.6(5.4) | 60 | 9.2(5.8) |
| **~8w post** | 8856 | 5.8(4.6) | 8529 | 5.7(4.6) | 122 | 7.7(5.0) | 148 | 7.2(4.9) | 57 | 8.6(6.5) |
| **~8m post** | 8578 | 5.2(4.5) | 8261 | 5.1(4.5) | 115 | 7.8(5.7) | 146 | 7.0(4.6) | 56 | 9.3(6.1) |
| **~2 years** | 8018 | 5.5(4.7) | 7733 | 5.5(4.7) | 105 | 7.9(5.1) | 129 | 7.1(5.3) | 51 | 7.9(5.5) |
| **~3 years** | 7572 | 6.1(4.9) | 7303 | 6.0(4.9) | 97 | 8.3(5.7) | 123 | 8.3(5.3) | 49 | 9.3(6.4) |
| **~5 years** | 7097 | 5.9(5.0) | 6837 | 5.8(4.9) | 95 | 7.7(5.5) | 117 | 8.2(5.5) | 48 | 8.5(5.3) |
| **~6 years** | 6864 | 6.2(5.1) | 6606 | 6.1(5.0) | 95 | 7.9(5.6) | 113 | 8.5(6.3) | 50 | 8.9(5.3) |
| **~8 years** | 6127 | 6.0(5.1) | 5904 | 5.9(5.1) | 79 | 8.4(5.4) | 99 | 8.2(5.8) | 45 | 10.5(6.5) |
| **~11 years** | 5867 | 5.6(5.2) | 5671 | 5.6(5.1) | 77 | 7.3(5.2) | 82 | 7.6(6.1) | 37 | 9.3(6.3) |
| **~18 years** | 3529 | 7.4(5.4) | 3387 | 7.3(5.4) | 56 | 9.3(5.7) | 56 | 8.2(4.8) | 30 | 11.5(7.4) |

**List of abbreviations:** w = weeks; m= months; gest = gestation; post = postnatal; SD = standard deviation

**Supplemental Table 3: Pairwise correlations of EPDS scores between each time point.**

| **Time point** | **18w gest** | **32w gest** | **~8w post** | **~8m post** | **~2 years** | **~3 years** | **~5 years** | **~6 years** | **~8 years** | **~11 years** | **~18 years** |
| --- | --- | --- | --- | --- | --- | --- | --- | --- | --- | --- | --- |
| **18w gest** | 1 |  |  |  |  |  |  |  |  |  |  |
| **32w gest** | 0.64 | 1 |  |  |  |  |  |  |  |  |  |
| **~8w post** | 0.53 | 0.57 | 1 |  |  |  |  |  |  |  |  |
| **~8m post** | 0.50 | 0.55 | 0.61 | 1 |  |  |  |  |  |  |  |
| **~2 years** | 0.49 | 0.52 | 0.56 | 0.62 | 1 |  |  |  |  |  |  |
| **~3 years** | 0.47 | 0.50 | 0.52 | 0.58 | 0.62 | 1 |  |  |  |  |  |
| **~5 years** | 0.44 | 0.46 | 0.46 | 0.50 | 0.54 | 0.58 | 1 |  |  |  |  |
| **~6 years** | 0.42 | 0.45 | 0.46 | 0.50 | 0.52 | 0.55 | 0.63 | 1 |  |  |  |
| **~8 years** | 0.41 | 0.42 | 0.42 | 0.45 | 0.50 | 0.52 | 0.57 | 0.58 | 1 |  |  |
| **~11 years** | 0.40 | 0.41 | 0.42 | 0.44 | 0.48 | 0.49 | 0.51 | 0.51 | 0.55 | 1 |  |
| **~18 years** | 0.36 | 0.38 | 0.40 | 0.40 | 0.44 | 0.43 | 0.47 | 0.45 | 0.46 | 0.49 | 1 |

|  | **Depressive symptoms** | | | | |
| --- | --- | --- | --- | --- | --- |
| **Fixed Effects** | **Model A**  **Coeff [95%CI]** | **Model B**  **Coeff [95%CI]** | **Model C**  **Coeff [95%CI]** | **Model D**  **Coeff [95%CI]** | **Model E**  **Coeff [95%CI]** |
| **Constant** | 6.19*** [6.12 ; 6.27] | 5.90*** [5.81 ; 5.99] | 6.93 [6.46 to 7.41] | 6.88 [6.40 to 7.35] | 6.86 [6.39 to 7.34] |
| **time** | 0.0034*** [0.0029 ; 0.0038] | -0.00051  [-0.0013 ; 0.00026] | -0.00043  [-0.0012 ; 0.00035] | -0.00043  [-0.0012 ; 0.00034] | -0.00063  [-0.0014 ; 0.00016] |
| **time^2^** |  | 0.000068***  [0.000060 ; 0.000076] | 0.000068***  [0.000060 ; 0.000076] | 0.000068***  [0.000060 ; 0.000076] | 0.000070***  [0.000063 ; 0.000078] |
| **Age at delivery** | |  |  |  |  |
|  |  |  | -0.025**  [-0.042 ; -0.0080] | -0.025**  [-0.042 ; -0.0082] | -0.025**  [-0.042 ; -0.0082] |
| **Social Class** |  |  |  |  |  |
| Manual |  |  | Ref | Ref | Ref |
| Non manual |  |  | -0.46*** [-0.65 ; -0.26] | -0.44*** [-0.63 ; -0.25] | -0.44*** [-0.63 ; -0.25] |
| **Education** |  |  |  |  |  |
| Compulsory |  |  | Ref | Ref | Ref |
| A Level or degree |  |  | -0.24** [-0.40 ; -0.076] | -0.27*** [-0.43 ; -0.11] | -0.27*** [-0.43 ; -0.11] |
| **Mental health problem in parents** | | | | | |
| No |  |  | Ref | Ref | Ref |
| Yes |  |  | 1.25*** [0.89 ; 1.61] | 1.17*** [0.81 ; 1.53] | 1.17*** [0.81 ; 1.53] |

**Supplemental Table 4: Full model results from longitudinal growth models on complete case sample (N = 9,276)**

**Supplemental Table 4: continued**

| **Depressive symptoms** | | | | | |
| --- | --- | --- | --- | --- | --- |
| **Fixed Effects** | **Model A**  **Coeff [95%CI]** | **Model B**  **Coeff [95%CI]** | **Model C**  **Coeff [95%CI]** | **Model D**  **Coeff [95%CI]** | **Model E**  **Coeff [95%CI]** |
| **Sexual abuse** |  |  |  |  |  |
| No |  |  | Ref | Ref | Ref |
| Yes |  |  | 1.95*** [1.59 ; 2.30] | 1.82*** [1.47 ; 2.17] | 1.82*** [1.47 ; 2.17] |
| **Lifetime eating disorder** | |  |  |  |  |
| *No ED* |  |  |  | Ref | Ref |
| *AN* |  |  |  | 1.88*** [1.24 ; 2.51] | 2.10*** [1.36 ; 2.83] |
| *BN* |  |  |  | 1.72*** [1.15 ; 2.30] | 2.28*** [1.61 ; 2.94] |
| *AN + BN* |  |  |  | 2.68*** [1.77 ; 3.60] | 2.86*** [1.81 ; 3.90] |
| **Lifetime Eating disorder** $\boldsymbol{\times}$**time** | |  |  |  |  |
| *No ED* |  |  |  |  | Ref |
| *AN* |  |  |  |  | 0.0034 [-0.0034 ; 0.010] |
| *BN* |  |  |  |  | 0.0073** [0.0012 ; 0.013] |
| *AN + BN* |  |  |  |  | 0.0054 [-0.0040 ; 0.015] |
| **Lifetime Eating disorder** $\boldsymbol{\times}$**time^2^** | |  |  |  |  |
| *No ED* |  |  |  |  | Ref |
| *AN* |  |  |  |  | -0.000037 [-0.00010 ; 0.000028] |
| *BN* |  |  |  |  | -0.00011*** [-0.00017 ; -0.000045] |
| *AN + BN* |  |  |  |  | -0.000016 [-0.00011 ; 0.000074] |
| **Goodness of fit parameters** | |  |  |  |  |
| AIC | 450365.3 | 447107.2 | 446874.3 | 446782.6 | 446780.1 |
| *BIC* | 450402.5 | 447200.1 | 447013.7 | 446949.9 | 447003.2 |

**Supplemental table 4: continued**

| **Depressive symptoms** | | | | | |
| --- | --- | --- | --- | --- | --- |
| **Random Effects** | **Model A**  **Coeff [SE]** | **Model B**  **Coeff [SE]** | **Model C**  **Coeff [SE]** | **Model D**  **Coeff [SE]** | **Model E**  **Coeff [SE]** |
| *Level 1 – Within individual* | 12.22***(0.065) | 10.43*** (0.061) | 10.4*** (0.06) | 10.4***(0.06) | 10.4***(0.06) |
| *Level 2 – Between individual* | 12.00*** (0.20) | 14.16*** (0.26) | 13.9*** (0.3) | 13.7*** (0.3) | 13.7*** (0.3) |
| **Linear term** |  |  |  |  |  |
| *Variance* |  | 0.00066*** (0.000021) | 0.0007*** (0.00002) | 0.0007*** (0.00002) | 0.0007*** (0.00002) |
| *Covariance (with intercept)* |  | 0.034*** (0.0018) | 0.03*** (0.002) | 0.03*** (0.002) | 0.03*** (0.002) |
| **Quadratic term** |  |  |  |  |  |
| *Variance* |  | 4.13x10^-08^ *** (1.97x10^-09^) | 4.1x10^-08^***(2.0x10^-09^) | 4.1x10^-08^***(2.0x10^-09^) | 4.1x10^-08^***(2.0x10^-09^) |
| *Covariance (with intercept)* |  | -0.00031*** (0.000018) | 0.0003***(0.00002) | -0.0003***(0.00002) | -0.0003***(0.00002) |
| *Covariance (with linear slope)* |  | -4.11x10^-06^ *** (1.71x10^-07^) | -4.1x10^-06^***(1.7x10^-07^) | -4.1x10^-06^***(1.7x10^-07^) | -4.1x10^-06^***(1.7x10^-07^) |

**0.01<p<0.05 **0.001<p<0.01 ***p<0.001*

**Supplemental Table 5: Results of growth curve models extended to all women with at least one outcome measurement. Complete case sample (N=9,536)**

|  | **Maternal depressive symptoms** | |
| --- | --- | --- |
|  | **Model D**  **Coeff [95%CI]** | **Model E**  **Coeff [95%CI]** |
| **Lifetime Eating disorder** |  |  |
| No | Reference | Reference |
| AN | 1.89 (1.27, 2.52), [p<0.0001] | 2.11 (1.38, 2.85), [p<0.0001] |
| BN | 1.72 (1.15, 2.29), [p<0.0001] | 2.28 (1.62, 2.94), [p<0.0001] |
| AN + BN | 2.81 (1.91, 3.71), [p<0.0001] | 2.98 (1.94, 4.01), [p<0.0001] |
| **Lifetime Eating disorder * time** |  |  |
| No | Reference | Reference |
| AN | - | 0.003 (-0.003, 0.01), [p=0.3359] |
| BN | - | 0.007 (0.001, 0.01), [p=0.0199] |
| AN + BN | - | 0.005 (-0.004, 0.01) , [p=0.2946] |
| **Lifetime Eating disorder * time^2^** |  |  |
| No | Reference | Reference |
| AN | - | -0.001 (-0.0001, 0.001), [p=0.2782] |
| BN | - | -0.001 (-0.0001, -0.001), [p=0.0006] |
| AN + BN | - | -0.001 (-0.0001, 0.001), [p=0.7549] |

**Supplemental Table 6: Results of sensitivity analysis based on a sample with women with complete exposure, at least 3 outcome measurements and imputed missing confounders and outcomes (N= 11,590). P values for random effects are unavailable as degrees of freedom could not be estimated from multiply imputed datasets.**

|  | **Maternal depressive symptoms** | | |
| --- | --- | --- | --- |
| **Fixed Effects** | **Model C**  **Coeff [95%CI]** | **Model D**  **Coeff [95%CI]** | **Model E**  **Coeff [95%CI]** |
| **Constant** | 7.19 [6.76 ; 7.62] | 7.15 [6.73 ; 7.59] | 7.15 [6.72 ; 7.58] |
| **time** | -0.00097**  [-0.0017 ; -0.00025] | -0.00097**  [-0.0017 ; -0.00025] | -0.0011**  [-0.0018 ; -0.00034] |
| **time^2^** | 0.000076***  [0.000068 ; 0.000083] | 0.000076***  [0.000068 ; 0.000083] | 0.000078***  [0.000070 ; 0.000085] |
| **Age** |  |  |  |
|  | -0.027***[-0.043 ; -0.012] | -0.029***[-0.044 ; -0.013] | -0.029*** [-0.044 ; -0.013] |
| **Social Class** |  |  |  |
| Manual | Ref | Ref | Ref |
| Non manual | -0.49***[-0.68 ; -0.29] | -0.47***[-0.67 ; -0.27] | -0.47***[-0.67 ; -0.27] |
| **Education** |  |  |  |
| Compulsory | Ref | Ref | Ref |
| A Level or degree | -0.35***[-0.51 ; -0.20] | -0.39***[-0.54 ; -0.23] | -0.39***[-0.54 ; -0.23] |
| **Mental health problem in parents** | |  |  |
| No | Ref | Ref | Ref |
| Yes | 1.18***[0.84 ; 1.53] | 1.09***[0.75 ; 1.44] | 1.09***[0.75 ; 1.44] |
| **Experience of sexual abuse** | |  |  |
| No | Ref | Ref | Ref |
| Yes | 2.14***[1.81 ; 2.47] | 2.01***[1.68 ; 2.34] | 2.01*** [1.68 ; 2.34] |
| **Lifetime Eating disorder** | |  |  |
| No | | Ref | Ref |
| AN | | 2.13***[1.55 ; 2.72] | 2.23***[1.57 ; 2.89] |
| BN | | 1.53***[0.993 ; 2.07] | 2.00***[1.38 ; 2.61] |
| AN + BN | | 2.99***[2.15 ; 3.82] | 3.08***[2.15 ; 4.01] |
| **Lifetime Eating disorder * time** | |  |  |
| No | |  | Ref |
| AN | |  | 0.00089 [-0.0051 ; 0.0069] |
| BN | |  | 0.0043 [-0.0016 ; 0.010] |
| AN + BN | |  | 0.0022 [-0.0060 ; 0.011] |
| **Lifetime Eating disorder * time^2^** | |  |  |
| No | |  | Ref |
| AN | |  | -0.000019 [-0.000075 ; 0.000036] |
| BN | |  | -0.000089**[-0.00015 ; -0.000032] |
| AN + BN | |  | -0.000015 [-0.000093 ; 0.000063] |

**Supplemental table 6: (continued, random effects)**

| **Random effects** | **Model C** | **Model D** | **Model E** |
| --- | --- | --- | --- |
| **Within individual variance (SE)** | 10.9(0.06) | 10.9(0.06) | 10.9(0.06) |
| **Between individual variance (SE)** | 14.2(0.2) | 14.0(0.2) | 14.0(0.2) |
| **Linear Term** |  |  |  |
| *Variance* | 0.0007 (0.00002) | 0.0006 (0.00002) | 0.0006 (0.00002) |
| *Covariance(with intercept)* | 0.03(0.002) | 0.03 (0.002) | 0.03 (0.002) |
| **Quadratic Term** |  |  |  |
| *Variance* | 3.7x10^-08^(2.0x10^-09^) | 3.7x10^-08^ (2.0x10^-09^) | 3.7x10^-08^(2.0x10^-09^) |
| *Covariance(with intercept)* | -0.0003(0.00001) | -0.0003(0.00001) | -0.0003(0.00001) |
| *Covariance(with linear slope)* | -3.8x10^-06^(1.6x10^-07^) | -3.8x10^-06^(1.6x10^-07^) | -3.8x10^-06^(1.6x10^-07^) |

*Model C = time + time^2^ + age + social class + education + parental mental disorder + sexual abuse*

*Model D = Model C + ED history*

*Model E = Model D + ED history x time + ED history x time^2^*

**0.01<p<0.05 **0.001<p<0.01 ***p<0.001*

**Supplemental Table 7: Association between lifetime self-reported ED and body image and eating concerns in pregnancy**

|  | **Crude model**  **Coefficient (95%CI)** | **Adjusted model**  **Coefficient (95% CI)** |
| --- | --- | --- |
| **Maternal self-reported ED** |  |  |
| None | Reference | Reference |
| AN | 1.15 (0.38 – 1.92), p=0.003 | 1.07 (0.30 – 1.84), p=0.006 |
| BN | 2.91 (2.23 – 3.60), p<0.0001 | 2.83 (2.14 – 3.51), p<0.0001 |
| AN + BN | 3.11 (2.03- 4.20), p<0.0001 | 2.89 (1.80 – 2.98), p<0.0001 |

Adjusted model: adjusted for maternal age, social class, education, history of sexual abuse, and parental mental health problems

**Supplemental Table 8: Sensitivity analysis model building**

|  | **Depressive symptoms** | | | | |
| --- | --- | --- | --- | --- | --- |
| **Fixed Effects** | **Model A**  **Coeff [95%CI]** | **Model B**  **Coeff [95%CI]** | **Model C**  **Coeff [95%CI]** | **Model D**  **Coeff [95%CI]** | **Model E**  **Coeff [95%CI]** |
| **Constant** | 6.14 [6.06, 6.22] | 5.87 [5.78, 5.96] | 6.97 [6.47, 7.46] | 5.73 [5.25, 6.23] | 5.84 [5.35, 6.34] |
| **time** | 0.003  [0.002, 0.003] | -0.0004  [-0.001, 0.0003] | -0.0004 [-0.002, 0.0004] | -0.0003 [-0.001, 0.0004] | 0.002 [0.00007, 0.003] |
| **time^2^** |  | 0.00007  [0.00006, 0.00008] | 0.00007  [0.00006, 0.00008] | 0.00007  [0.00006, 0.00008] | 0.00005  [0.00004, 0.00007] |
| **Age** |  |  |  |  |  |
|  |  |  | -0.03 [-0.05, -0.01]** | -0.03 [-0.05, -0.01]** | -0.03 [-0.05, -0.01]** |
| **Social Class** |  |  |  |  |  |
| Manual |  |  | Ref | Ref | Ref |
| Non manual |  |  | -0.44 [-0.64, -0.24]*** | -0.41 [-0.60, -0.13]*** | -0.41 [-0.60, -0.22]*** |
| **Education** |  |  |  |  |  |
| Compulsory |  |  | Ref | Ref | Ref |
| A Level or degree |  |  | -0.29 [-0.46, -0.13]** | -0.26 [-0.42, -0.11]** | -0.27 [-0.42, -0.11]** |
| **Mental health problem in parents** | | | | | |
| No |  |  | Ref | Ref | Ref |
| Yes |  |  | 1.16 [0.79, 1.52]*** | 1.03 [0.68, 1.38]*** | 1.03 [0.68, 1.38]*** |

| **Depressive symptoms** | | | | | |  |
| --- | --- | --- | --- | --- | --- | --- |
| **Fixed Effects** | **Model A**  **Coeff [95%CI]** | **Model B**  **Coeff [95%CI]** | **Model C**  **Coeff [95%CI]** | **Model D**  **Coeff [95%CI]** | **Model E**  **Coeff [95%CI]** |  |
| **Sexual abuse** |  |  |  |  |  |  |
| No |  |  | Ref | Ref | Ref |  |
| Yes |  |  | 1.81 [1.44, 2.17]*** | 1.50 [1.15, 1.85]*** | 1.50 [1.15, 1.85]*** |  |
| **Lifetime eating disorder** | |  |  |  |  |  |
| *No ED* |  |  | Ref | Ref | Ref |  |
| *AN* |  |  | 1.78 [1.07, 2.40]*** | 1.50 [0.86 ;2.13]*** | 1.49 [0.86 ; 2.13]*** |  |
| *BN* |  |  | 1.88 [1.28, 2.47]*** | 1.16 [0.59 ; 1.73]*** | 1.16 [0.59 ; 1.73]*** |  |
| *AN + BN* |  |  | 2.50 [1.56, 3.45]*** | 1.84 [0.94 ;2.74]*** | 1.84 [0.94 ;2.74]*** |  |
| **Quintiles of body image and eating concerns** | |  |  |  |  |  |
| *1^st^ (lowest)* |  |  |  | Ref | Ref |  |
| *2^nd^* |  |  |  | 0.75 [0.55 ; 0.97]*** | 0.68 [0.43 ; 0.92]*** |  |
| *3^rd^* |  |  |  | 1.20 [0.98 ; 1.42]*** | 1.15 [0.89 ; 1.41]*** |  |
| *4^th^* |  |  |  | 1.94 [1.72 ; 2.15]*** | 1.78 [1.52 ; 2.04]*** |  |
| *5^th^ (highest)* |  |  |  | 3.23 [3.01 ;3.46]*** | 2.88 [2.62 ; 3.14]*** |  |
| **Quintiles of body image and eating concerns**$\boldsymbol{\times}$**time** | |  |  |  |  |  |
| *1^st^ (lowest)* |  |  |  |  | Ref |  |
| *2^nd^* |  |  |  |  | -0.001 [-0.003 ; 0.002] |  |
| *3^rd^* |  |  |  |  | -0.001 [-0.004 ; 0.001] |  |
| *4^th^* |  |  |  |  | -0.003 [-0.005 ; -0.001]* |  |
| *5^th^ (highest)* |  |  |  |  | -0.006 [-0.009 ; -0.004]*** |  |
| **Quintiles of body image and eating concerns** $\boldsymbol{\times}$**time^2^** | |  |  |  |  |  |
| *1^st^ (lowest)* |  |  |  |  | Ref |  |
| *2^nd^* |  |  |  |  | 0.00002 [-0.000006 ; 0.0003] |  |
| *3^rd^* |  |  |  |  | 0.000003 [-0.00002 ; 0.00003] |  |
| *4^th^* |  |  |  |  | 0.00002 [-0.000002 ;0.00005] |  |
| *5^th^ (highest)* |  |  |  |  | 0.00005 [0.00003 ; 0.00008]*** |  |
| **AIC** | 425607.4 | 424148.5 |  | 422997.2 | 422980.2 |  |
| **BIC** | 425662.9 | 424141.0 |  | 423200.5 | 423257.5 |  |

**Supplemental Figure 1. Predicted trajectories of depressive symptoms derived from Model E** **by quintiles of eating disorder cognitions in pregnancy**


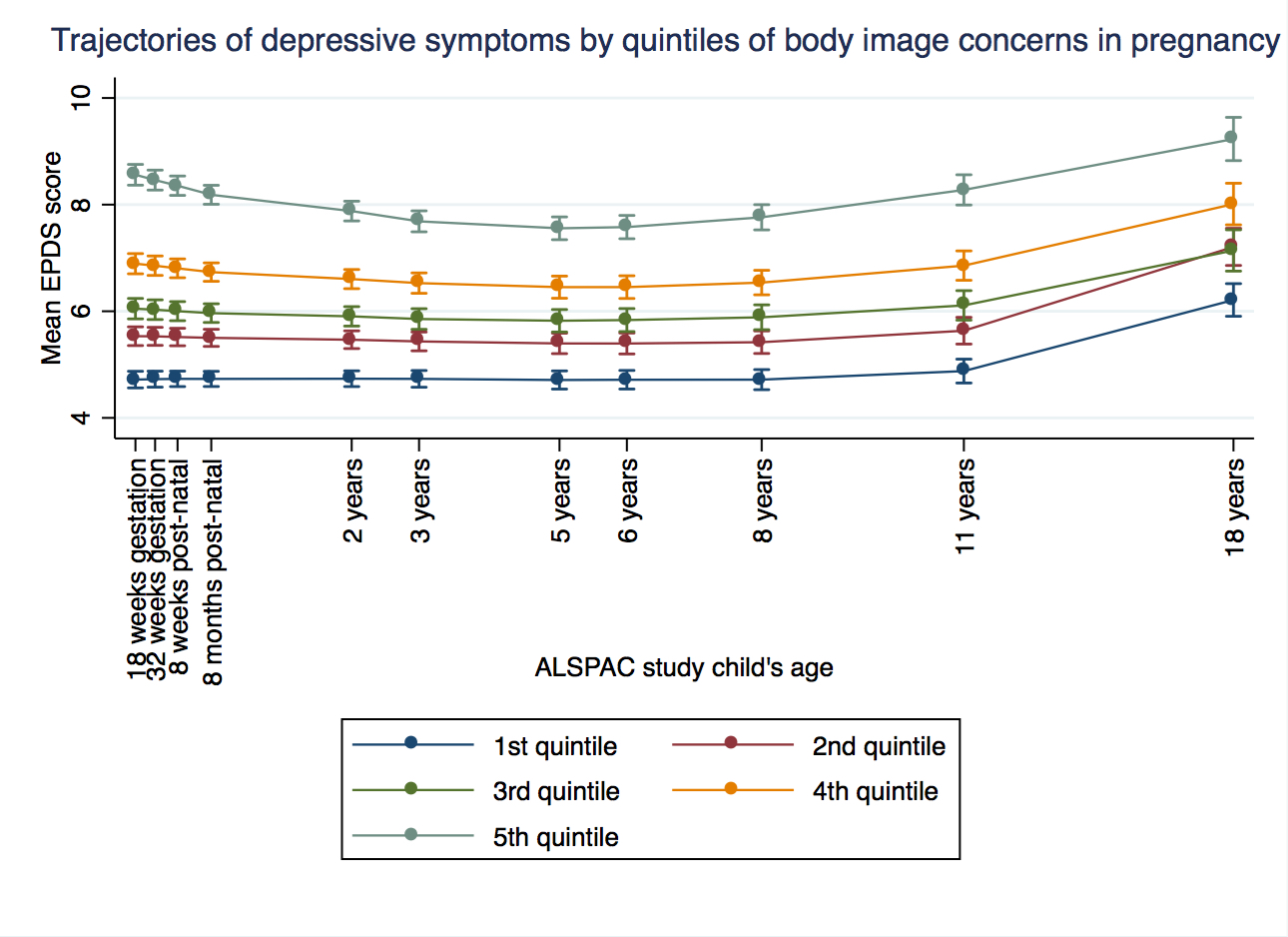

Supplement: Supplementary file 1 [file S0007125019000898sup001.docx]
